# Anti-Obesity Effect of Combining White Kidney Bean Extract, Propolis Ethanolic Extract and CrPi_3_ on Sprague-Dawley Rats Fed a High-Fat Diet

**DOI:** 10.3390/nu16020310

**Published:** 2024-01-20

**Authors:** Doaa Salah Eldin Abdelfattah, Mervat A. Fouad, Aliaa N. Elmeshad, Mohamed A. El-Nabarawi, Sammar Fathy Elhabal

**Affiliations:** 1 National Nutrition Institute, Cairo 11435, Egypt; mevo_73@hotmail.com; 2 Department of Pharmaceutics and Industrial Pharmacy, Faculty of Pharmacy, Cairo University, Giza 11562, Egypt or anabil@ecu.edu.eg (A.N.E.); mohamed.elnabarawi@pharma.cu.edu.eg (M.A.E.-N.); 3 Department of Pharmaceutics, Faculty of Pharmacy and Drug Technology, The Egyptian Chinese University, Cairo 11786, Egypt; 4 Department of Pharmaceutics and Industrial Pharmacy, Faculty of Pharmacy, Modern University for Technology and Information (MTI), Cairo 11571, Egypt; sammar.fathy@pharm.mti.edu.eg

**Keywords:** obesity, metabolic disease, bioactive components, white kidney bean extract, propolis ethanolic extract, CrPi_3_, nutraceutical, α-amylase, flavonoids, polyphenols

## Abstract

Obesity has been associated with the occurrence and prevalence of various chronic metabolic diseases. The management of obesity has evolved to focus not only on reducing weight, but also on preventing obesity-related complications. Studies have shown that bioactive components in natural products like white kidney bean extract (WKBE), propolis ethanolic extract (PEE), and chromium picolinate (CrPi_3_) showed anti-obesity properties. However, no studies have examined the outcomes of combining any of these nutraceutical supplements. We compared the effects of HFD supplemented with WKBE, WKBE+PEE, or WKBE+PEE+CrPi_3_ against control and obese groups using Sprague-Dawley rats fed a 45% high-fat diet as an in vivo model. Nutritional parameters, biochemical parameters, and biomarkers of cardiovascular disease, liver function, kidney function, and gut health were among the comparable effects. Our findings showed that combining the three nutraceutical supplements had a synergetic effect on reducing weight gain, food utilization rate, abdominal fat, serum lipids, arterial and hepatic lipids, risk of cardiovascular disease, and blood glucose level, in addition to improving renal function and gut microbiota. We attributed these effects to the α-amylase inhibitor action of WKBE, flavonoids, and polyphenol content of PEE, which were potentiated with CrPi_3_ resulting in a further reduction or normalization of certain parameters.

## 1. Introduction

A World Health Organization (WHO) report from 2021 states that in 2016, more than 650 million adults were obese and over 1.9 billion individuals were overweight, with the proportion of obese people having nearly quadrupled between 1975 and 2016 [1]. Obesity is a global public health concern not only because it lowers people’s quality of life, but also because it acts as a proxy for many comorbidities, making it the leading cause of preventable death globally [2,3]. It is the second biggest risk factor after smoking, accounting for up to 3.4 million deaths annually [1]. Epidemiological research has connected obesity to a higher risk of cardiovascular disease, type 2 diabetes, dyslipidemia, sleep apnea, knee osteoarthritis, and some types of cancer [4,5,6,7]. In the wake of the COVID-19 pandemic, obesity has been found to be a major risk factor for hospitalization and subpar clinical outcomes for SARS-CoV2 patients [8]. Although its pathophysiology is very complicated, it is influenced by both congenital (inherited) and acquired (environmental) variables [3]. Numerous factors contribute to the risk of obesity, such as an increase in sedentary activity and unhealthy eating patterns, such as consuming more foods high in saturated fats and refined sugars, and low in fiber [9,10]. Consequently, obesity is caused by a persistent imbalance between energy expenditure and intake, which results in aberrant fat storage in adipose tissues, improper lipid metabolism, and excessive insulin production, which induces insulin resistance [11].

Obesity treatment aims not only to reduce weight but also to reduce obesity-related complications. Treatment options for obesity vary widely and include calorie restriction paired with increased physical activity, weight loss surgery, prescription weight-loss medicines, and lifestyle adjustment [12]. If provided with ways to achieve quick weight loss, obese patients may feel more motivated to alter their lifestyle choices and more confident in their ability to reach their objectives. Although anti-obesity medications may seem like a promising way to speed up weight loss, their potential side effects and drug interactions highlight the need to find novel, efficient, and safe anti-obesity management plans. 

Nowadays, people choose to use natural products due to their efficaciousness in treating obesity and several other chronic illnesses. As an alternative to current methods of treating obesity, there is growing interest in the utilization of natural extracts and bioactive components from plants, such as fruits, vegetables, cereals, and herbs. The most widely used strategy in the functional supplement market is creating dietary supplements using everyday foods [13,14]. It has been discovered that dietary supplements containing phytochemicals, polyunsaturated fatty acids, and dietary fibers have anti-obesity effects through a variety of routes, including providing satiety, energy expenditure, inhibition of adipocyte differentiation, and lipid synthesis enzymes [13,14,15].

Due to its well-established anti-obesity properties, the white kidney bean (*Phaseolus vulgaris* L.), one of the traditional legumes in South America and Africa, is gaining more and more attention on a global scale [16]. White kidney bean extract is particularly interesting because it contains protein-based α-amylase inhibitor (α-AI), which functions as a “starch blocker” by binding to the target enzyme, α-amylase, which is an essential enzyme for the digestion of carbohydrates. The hydrolysis of starch in food is hindered and the digestion of large carbohydrates is reduced when the amylase enzyme is inhibited [17]. White kidney bean extract is a promising anti-obesity candidate that controls fat buildup, lowers postprandial plasma hyperglycemia and insulin, and has anorexigenic effects, according to numerous in vivo investigations conducted in both animal and human models [18].

Propolis is another natural substance that has gained attention because of its diverse biological characteristics. Since ancient times, propolis, a naturally occurring product obtained from beekeeping, has been utilized in folk medicine. Numerous pharmacological effects have been related to propolis, including immune modulatory, antitumoral, antibacterial, antiviral, antifungal, anti-inflammatory, and antioxidant qualities [19]. Propolis’s composition changes depending on its source, location, time of year, colonies, and the sections of the plant that bees may access [19]. In general, 50–55% of propolis is composed of resin (esters, phenolic acids, and flavonoids), in addition to volatile compounds (10%), beeswax (30–40%), pollen (5–10%), and other materials [20]. Different botanical varieties of propolis exist depending on where it originates from, but Poplar type, Baccharis type, and Dalbergia type are the three types of propolis with anti-obesity impact [21]. Egyptian propolis is a member of the Poplar-type botanical source family, which is widely found in North Africa, much of Asia, Europe, and North America [22]. Propolis’s high polyphenol content has been linked to remarkable anti-obesity efficacy, according to numerous in vivo and in vitro studies [19,21]. According to these study findings, propolis extract improves lipid metabolism and reduces insulin resistance to regulate weight reduction [23]. Furthermore, in rats with induced diabetes mellitus and insulin resistance, ethanol-extracted propolis was observed to modulate blood glucose, glucose metabolism, and blood lipid concentration [24].

Chromium (Cr^3+^) is a widely distributed important ingredient in the human diet that is needed for insulin function as well as the proper metabolism of proteins, fats, and carbohydrates. According to current dietary recommendations, adults should consume between 25 and 45 μg of chromium per day, and between 1% and 2% of chromium is thought to be absorbed through the diet [25]. Chromium (III) picolinate (CrPi_3_) is a trivalent chromium compound, complexed with picolinic acid, a natural derivative of tryptophan [26]. Chromium dietary supplements have improved insulin sensitivity and glucose metabolism, which has reduced blood glucose levels in overweight diabetics. It has been suggested that the mechanism of action involves a decrease in body fat and actions that make insulin more sensitive by increasing insulin activity or upregulating insulin receptors, both of which could help in the management of weight [27,28]. CrPi_3_ might have an influence on the neurotransmitters that control eating habits, inhibit appetite, lessen food cravings, improve thermogenesis, raise resting energy expenditure, and decrease hunger, all of which may lead to a decrease in caloric intake, according to earlier research [29].

Our research aimed to formulate and determine the effectiveness of combining the bioactive substance in the three nutraceutical supplements containing white kidney bean extract (WKBE), propolis ethanolic extract (PEE), and CrPi_3_ in an obesity-induced rat model and compare their anti-obesity effects with normal and obese rats. Comparable effects included nutritional parameters, biochemical parameters, and biomarkers of cardiovascular disease, liver function, and kidney function. The research question was whether these parameters would be differently affected as a result of incorporating any of the three formulated supplements, and which supplement would be the most efficient in reverting or normalizing HFD-induced obesity and its associated side effects. The hypothesis was that combining PPE with WKBE would have synergistic effects; however, adding CrPi_3_ can lead to overall additional benefits. Our findings showed that the best results were achieved upon combining the three nutraceutical supplements, where effects including reduced weight gain, food utilization rate, abdominal fat, serum lipids, hepatic and arterial lipids, blood glucose level, risk of cardiovascular disease, and improved gut microbiota and renal function were reported. Despite the fact that several studies have examined the effects of administering a diet containing either WKBE, PPE, or CrPi_3_ on body weight and the relative weight of organs in comparison to either a basal diet and/or HFD. To the best of our knowledge, our research is the first to combine these three nutraceutical supplements, WKBE, PPE, and CrPi_3_ to supplement a high-fat diet and compare their effects against both a basic diet (as a negative control) and HFD (as a positive control). This work contributes to summarizing the knowledge and application of different nutraceutical formulas for controlling obesity and also highlights the potential outcome of combining nutraceutical supplements to optimize their overall anti-obesity effects.

## 2. Materials and Methods

### 2.1. Materials

White kidney bean (*Phaseolus vulgaris*) extract (WKBE) was obtained from Xi’an Sentian Biotechnology Co. Ltd. (Shanghai, China). Egyptian propolis was purchased from a local natural product store in the greater Cairo area of Egypt. Food grade chromium picolinate (elemental chromium of 12.5%) was obtained from El-Gomhoria Company, Cairo, Egypt. Commercial assay kits supplied by Bio Diagnostic, Egypt were used for the analysis of total cholesterol (TC), triglyceride concentration (TG), total phospholipids, high-density lipoprotein cholesterol (HDL-C), malonaldehyde (MDA), liver enzymes (AST, ALT, and ALP), and kidney function biomarkers (creatinine, uric acid, urea). A glucose kit was purchased from HUMAN (Wiesbaden, Germany). Casein, vitamins, minerals, cellulose, sucrose, choline bitartrate, food grade chromium picolinate and tert-butylhydroquinone were purchased from El-Gomhoria Company, Cairo, Egypt. Soybean oil and palm oil were purchased from a local market in Cairo, Egypt. Corn starch and dextrinized corn starch were obtained from Starch and Glucose Manufacturing Co., Cairo, Egypt. Analytical chemicals, solvents, and standards were obtained from Sigma-Aldrich (St. Louis, MI, USA).

### 2.2. Determination of the α-Amylase Inhibitory Activity (α-AIE) in the White Kidney Bean Extract (WKBE)

White kidney bean (*Phaseolus vulgaris* L.) extract (WKBE) was characterized using a method previously reported by [30] where one inhibitory unit was represented by the amount of α-amylase inhibitor that totally inhibited the activity of one unit of enzyme.

### 2.3. Propolis Source and Extraction

Egyptian propolis was collected from a local natural product store specializing in natural honeybees and their products in the great Cairo region of Egypt. It was stored at −20 °C prior to extraction. The latter was preformed according to [31].

### 2.4. Estimation of Total Concentration of Phenols in PEE

The Folin–Ciocâlteu colorimetric test was used to determine the total phenol concentration in the propolis ethanolic extract, as described by [31]. The experiment was performed in triplicate, and then a spectrophotometer was used to measure absorbance at λ = 765 nm using this formula:

C = C1 × (V/m)(1)

where:

C = total concentration of phenols in mg/g, in GAE (Gallic acid equivalent), 

C1 = Gallic acid concentration determined from the calibration curve in mg/mL, 

V = extract volume in mL, and

m = plant extract weight in g.

### 2.5. Animals and Experimental Diets

Forty-five healthy male Sprague-Dawley rats weighing (160 ± 10 g) were acquired from the laboratory animal colony at the Helwan Farm Vaccine and Immunity Organization, Ministry of Health, Cairo, Egypt. The rats were handled in accordance with the experiment procedures approved by the Institutional Ethics Committee of Faculty of Pharmacy Cairo University, Cairo, Egypt (protocol code 1143) for studies involving animals to minimize animal suffering. The rats were kept in a 12-h light/dark cycle in individual stainless steel cages in an animal room with a controlled relative humidity (50%) and temperature of 24 °C. The rats were given a conventional laboratory diet and unlimited access to tap water during the acclimation period. The rats were given free access to food and water for the duration of the 14-week trial. 

Following a 7-day period of acclimation, rats were allocated at random to two groups: G1 (control group, *n* = 9) and high-fat diet (HFD) group, *n* = 36. For 6 weeks, the rats in the G1 group were fed a basic diet, while the 36 rats in the other group were fed a high-fat diet (HFD) that contained 45% calories from fat in order to develop obesity. The main sources of fat were soybean oil and lard.

**Figure 1:**
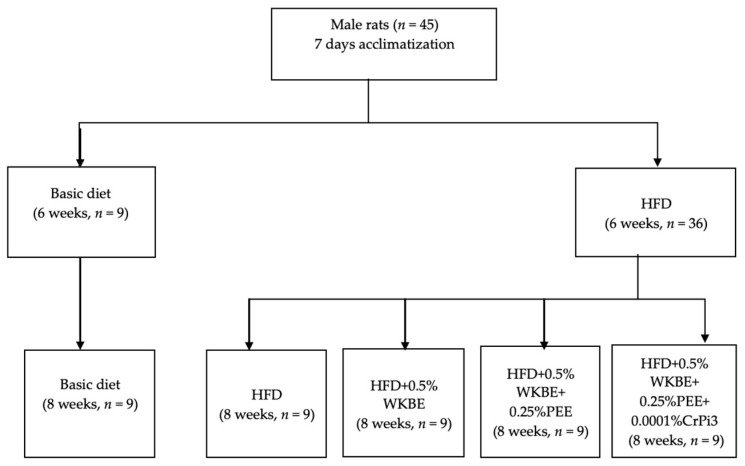
Experiment design and allocation of rats to diet groups.

After the 6-week duration, the control group G1 (*n* = 9) continued to feed on a basic diet while the 36 rats in the HFD group were assigned at random to one of the four groups listed below (Figure 1):

G2 (*n* = 9): HFD induced-obese group (+ve control)

G3 (*n* = 9): Supplement I [HFD + 0.5% WKBE]

G4 (*n* = 9): Supplement II [HFD + 0.5% WKBE + 0.25% PEE]

G5 (*n* = 9): Supplement III group [HFD + 0.5% WKBE + 0.25% PEE + 0.0001%CrPi_3_]

The second duration of the study was 8 weeks, where rats in the five groups (G1, G2, G3, G4, and G5) were fed their allocated diet. 

All diets were prepared using purified ingredients according to the animal feed formula of the American Institute of Nutrition (AIN-93G) [32]. The experimental and control diet compositions are shown in (Table 1) as (g/1000 g diet).

### 2.6. Measurement of Body Weight Gain, Food Consumption, and Food Utilization Rate

The rats’ food consumption and body weight increase were tracked weekly throughout the trial. Total food consumption was determined as the difference between the weekly food provided (g) versus the recovered weight of food (g). The formula for calculating the average food utilization rate (%) was as follows:

Average food utilization rate (%) = (total body weight gain/total food consumption) × 100%.(2)

### 2.7. Collection of Blood Samples and Analysis

At the end of the 14 weeks of study and following an overnight fast, the rats were anesthetized with diethyl ether and sacrificed, and the following samples were collected for analysis following the manufacturer’s instructions of the commercial kits from Bio Diagnostic, Egypt.

After drawing blood samples from the aorta (5 mL) in nonheparinized vacuum collection tubes, blood was left at room temperature for 15–30 min to allow it to clot, and then centrifuged at 2000 rpm for 10 min at 4 °C to separate the serum into an Eppendorf tube. The following parameters were measured in the serum: total cholesterol (TC) using the enzymatic method according to [33], triglyceride concentration (TG) using the calorimetric method as indicated by [34], the total phospholipid enzymatic method as described by [35], and high-density lipoprotein cholesterol (HDL-C) using the precipitation technique as outlined by [36].

Low-density lipoprotein cholesterol (LDL-C) [37] was calculated using the following formula:

LDL-C (mmol/L) = (TC) − (HDL-C + TG/5).(3)

Very low-density lipoprotein (VLDL-C) in the serum was calculated according to [38] by applying the formula outlined below:

VLDL-C (mmol/L) = TG/5(4)

The atherogenic index (AI) in the serum was calculated according to [39] by applying the formula outlined below:

AI = [TC − HDL-C/HDL-C](5)

Finally, the coronary risk ratio (CRR) was calculated applying the formula that outlined below:

Coronary risk ratio (CRR): CRR = [TC/HDL-C](6)

Separated serum samples were used to evaluate the hepatic and renal indicators. Measurements of alanine transaminase activity (ALT) and aspartate transaminase (AST) activities were enzymatically assessed according to [40], while serum alkaline phosphate (ALP) was estimated colorimetry using p-nitrophenyl phosphate according to the method outlined by [41]. Urea was measured according to [42]. Serum creatinine was colorimetrically determined by Jaffe’s reaction according to [43], while uric acid was determined using the enzymatic colorimetric method as described by [44]. The method outlined by [45] was used to measure malondialdehyde (MDA) in serum samples using kits available from the Bio Diagnostic Chemical Company (Egypt). The blood sugar level was estimated using a glucose kit from HUMAN (Germany) according to [46].

### 2.8. Weighing of Organs and Determination of Hepatic, Arterial, and Fecal Lipid Content

After being removed and washed in a cold saline solution, the kidneys, liver, heart, aorta, spleen, and abdominal fat were blotted on filter paper and weighed separately in order to determine their respective weights. The relative weight of organs was calculated as follows:

Relative weight of organs (%) = (Organ weight/body weight) × 100(7)

The liver and aorta were stored at −40 °C until further lipid analysis, while the entire colon was extracted. Its content was emptied then stored at −40 °C until further lipid analysis. Tissue samples were homogenized with 10 mL water, and 2 mL of the homogenate was mixed with 5 mL of Folch solution (chloroform–methanol ratio 2:1) by volume. Samples were vigorously shaken, then centrifuged at 3000 rpm for 5 min. The lower chloroform phase was collected and centrifuged at 3000 rpm for 5 min. The lower phase, which contained the lipids, was collected again, filtered, and dried at 80 °C to vaporize the solvents, and then the lipid content was measured by using the enzymatic kits. The total arterial cholesterol, triglycerides, and phospholipids as well as total hepatic lipids, cholesterol, and triglycerides and fecal lipids were analyzed according to previously reported methodology [47]. 

### 2.9. Determination of the Colonic Content pH, SCFA Concentration and Profile

The colonic contents were defrosted at 4 °C, homogenized, then diluted 10 times (*w*/*v*) in distilled water. A pH meter was used to record the colonic content pH values. The SCFA concentration was determined in accordance with [48]. 

### 2.10. Statistics 

The means ± standard error (SE) was used to express all data. One-way analysis of variance (ANOVA) was utilized to identify the differences among various dietary groups, and differences within treatments were analyzed post hoc by the least significant difference (LSD). Statistical Package for the Social Sciences (SPSS) 28.0 software was used to conduct the statistical studies. A significance threshold of *p* < 0.05 was applied to differences [49].

## 3. Results

### 3.1. Determining the α-Amylase Inhibitory Activity of White Kidney Bean Extract (WBE), and the Total Phenolic and Flavonoid Contents of the Propolis Ethanolic Extract (PEE)

The α-amylase inhibitory activity of WKBE against porcine pancreatic amylase was evaluated to be 3000 U/g. Propolis showed strong antioxidant activity. In particular, propolis extract had a total phenolic content of 137.32 ± 3.16 mg gallic acid/g and a total concentration of flavonoid of 41.6 ± 0.68 mg quercetin/g. 

### 3.2. Treatments and Measurement of the Body Weight Gain, Food Consumption, and Food Utilization Rate

**Figure 2:**
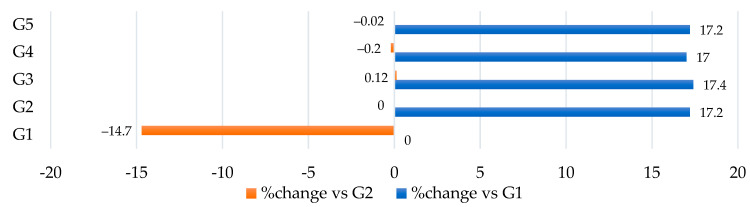
Initial weight of the Sprague-Dawley rats following 6 weeks of a basic diet, HFD, and different nutraceutical supplements.

The effects of the three anti-obesity nutraceutical supplements were assessed using a high-fat diet-induced rat model. Sprague-Dawley rats were fed for 18 weeks with either a basic diet representing the control group (G1), a high-fat diet (HFD) representing positive control (G2) induced-obese group, supplement I (HFD–WKBE) representing (G3), supplement II (HFD–WKBE–PEE) representing (G4), or supplement III (HFD–WKBE–PEE–CrPi_3_) representing (G5). Throughout the entire study, the positive control rats were kept as a point of reference to show that feeding on a high-fat diet did, in fact, cause obesity and its symptoms, including hyperlipidemia and an increased risk of cardiovascular disease. The initial weights of the rats in each of the five groups were recorded following the first 6 weeks as shown in Table 2, which demonstrated a significant change (*p* < 0.05) of 17.2%, 17.4%, 17%, and 17.2%, among G2, G3, G4 and G5, respectively, greater than the control group (G1) as shown in (Figure 2), suggesting that high-fat diet-induced obesity in these rats added to their body weight gain.

**Figure 3:**
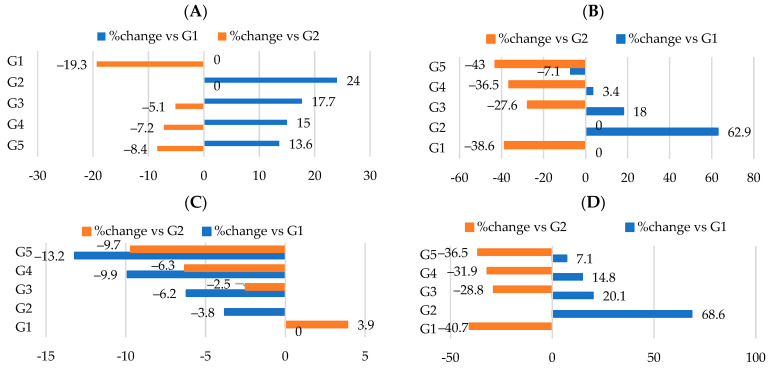
Percentage of nutritional changes among all groups. (**A**) Final body weight. (**B**) Body weight gain. (**C**) Food consumption. (**D**) Food utilization rate (%).

After 18 weeks, at the end of the study, the final weights were recorded (Table 2) and the rats in G2 showed a significant (*p* < 0.05) increase in their final weight with a percentage of increase of 24% compared to the control group (Figure 3A). As anticipated, the final weights for G3, G4, and G5 were reduced significantly compared to the positive control group (G2) with percentage changes of −5.1%, −7.2%, and −8.4%, respectively (Table 2, Figure 3A). G5 showed the least body weight gain followed by G4 then G3 groups (Table 2, Figure 3B). The food consumption of G3 showed an insignificant decrease (*p* > 0.05) compared to G2; however, G4 and G5 values were significantly reduced (*p* < 0.05) (Table 2, Figure 3C). The food utilization rate was determined by multiplying body weight gain by food consumption. Our data showed significant (*p* < 0.05) differences in food utilization rates between the G3, G4, and G5 groups, with rats in the G3 group having the highest rate, followed by G4 and then G5 (Table 2, Figure 3D) when compared to G2.

Overall, there was a decrease in body weight gain, food consumption, and food utilization rate when supplementing HFD with WKBE compared to the obese group (G2), with percentage changes of −27.6% (Figure 3B), −2.5% (Figure 3C), and −28.8% (Figure 3D), respectively. Simultaneously, adding PEE and CrPi_3_ to HFD−WKBE demonstrated additional advantages and significantly enhanced (*p* < 0.05) the values of the body weight gain when compared with WKBE alone. This was demonstrated by the percentage changes in body weight gain of G4 and G5 (−36.5% and −43%, respectively) (Figure 3B), food consumption (−6.3% and −9.7%, respectively) (Figure 3C), and food utilization rate (−31.9% and−16.5%) (Figure 3D).

### 3.3. Effect of Different Nutraceutical Supplements on Relative Organs and Tissue Weight

**Figure 4:**
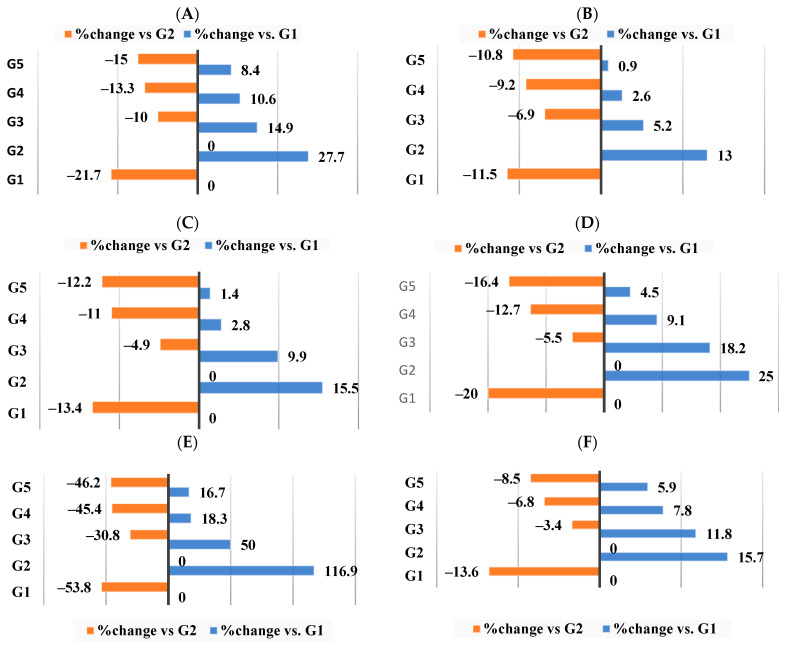
Percentage (%) of changes in relative weight of organs among all groups. (**A**) Relative weight of liver. (**B**) Relative weight of kidney. (**C**) Relative weight of spleen. (**D**) Relative weight of heart. (**E**) Relative weight of aorta. (**F**) Relative weight of intra-abdominal fat.

The relative weights of the organs changed in conjunction with the change in body weight. The effect of the various supplements on the relative weight of the organs and the accumulation of intra-abdominal fat is displayed in (Table 3) where the relative weight of organ = [(organ weight/body weight) × 100]. According to our findings, G2 demonstrated a marked increase in the relative weights of the liver, spleen, kidney, heart, aorta, and intra-abdominal fat following the 18-week experiment, as a result of induced obesity. However, the relative weights of the organs were significantly (*p* < 0.05) improved by co-treatment with WKBE alone, PEE alone, or in combination with CrPi_3_. Supplement III for G5 demonstrated the best recovery when compared to G2, but supplement II for G3 and supplement III for G4 showed a normalization in the relative weight of the kidney and spleen to values that were insignificantly different (*p* > 0.05) from the control group (G1) (Table 3). As shown in (Figure 4A–F), the administration of HFD extract showed a significance increase (*p* < 0.05) in the relative weight of organs including the liver, kidney, spleen, heart, aorta and intra-abdominal fat with percentages changes of 27.7%, 13%, 15.5%, 116.9%, 25%, and 15.7%, respectively. This effect was reversed by administering the HFD−WKBE, where the relative weights of these organs were decreased with a percentage change of −10%, 14.9%, −6.9%, −4.9%, 30.8%, −5.5% and −3.4%, respectively. Adding PEE only to the G4 supplement resulted in improvements in these results, where the relative weights of the liver decreased with a percentage change of (−13.3%), kidney (−9.2%), spleen (−11%), heart (−12.7%), aorta (−45.5%), and intra-abdominal fat (−6.8%). When CrPi_3_ was added to the G5 supplement, the percentage change in the relative weights of the liver (−15%), kidney (−10.8%), spleen (−12.2%), heart (−16.4%), aorta (−46.2%), and intra-abdominal fat (−8.5%) was further attenuated.

### 3.4. Effect of Different Nutraceutical Supplements on Lipid Profiles

**Figure 5:**
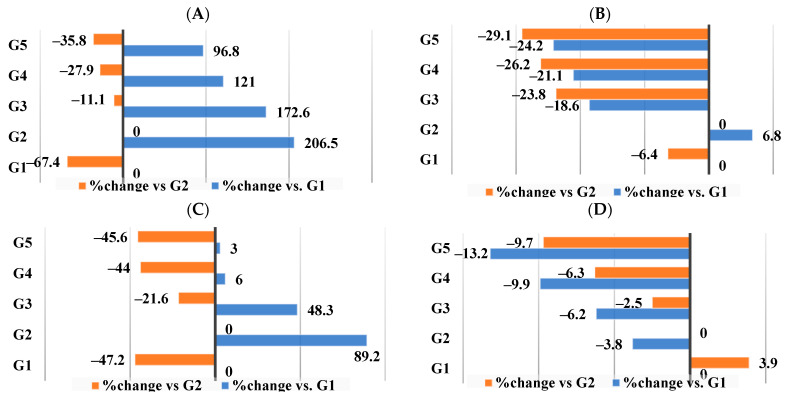

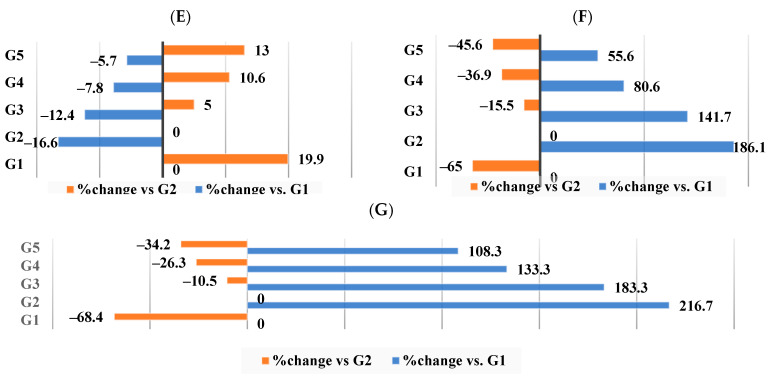
Percentage (%) of changes in lipid profile in blood. (**A**) Triglycerides. (**B**) Phospholipids. (**C**) Malondialdehyde. (**D**) Total cholesterol. (**E**) HDL-C. (**F**) LDL-C. (**G**)VLDL-C.

Table 4 and Figure 5A–G show the effect of WKBE, PEE, and CrPi_3_ on lipid profiles, atherogenic indices, and coronary risk ratios. It also reveals that the group receiving G2 had developed the hallmark features of dyslipidemia, including significantly (*p* < 0.05) elevated phospholipids, TC, TG, LDL-C, and VLDL-C levels, as well as a reduced HDL-C level compared to the control group. Conversely, G3 demonstrated a significant decrease (*p* < 0.05) in their lipid profile, which included TG, phospholipids, TC, LDL-C, and VLDL-C, along with a significant rise (*p* < 0.05) in HDL-C levels. In addition, G4 showed significantly lower (*p* < 0.05) levels of TG, phospholipids, TC, LDL-C, VLDL-C, and HDL-C when compared to G2, the group that was induced to become obese. Additionally, there was a significant rise (*p* < 0.05) in HDL-C and a further significant decrease (*p* < 0.05) in these levels in the G5 treatment with supplement III (HFD–WKBE–PEE–CrPi_3_). These findings were consistent with significantly lower LDL-C/HDL-C ratios, atherogenic indices, and coronary risk ratios among G3, G4, and G5. The same pattern was seen in the levels of malondialdehyde (MDA), which significantly increased (*p* < 0.05) in G2 relative to the control group, while the other groups experienced significant decreases (*p* < 0.05), with G5 showing the most reduced value.

As seen in Figure 5A–G, our data showed that supplement III (G5), which included CrPi_3_ added to the HFD–WKBE–PEE diet, demonstrated the best results when comparing the percentage of changes in serum lipid profile parameters among G3, G4, and G5 in comparison to G2. TG (−35.8%) (Figure 5A), phospholipid (−29.1%) (Figure 5B), MDA (−45.6%) (Figure 5C), TC (−9.7%) (Figure 5D), HDL-C (13%) (Figure 5E), LDL-C (−45.6%) (Figure 5F), and VLDL-C (−34.2%) (Figure 5G) are the percentage changes in lipids among the rats in G5.

### 3.5. Effect of Different Nutraceutical Supplements on Total Artery Cholesterol, Triglycerides, Phospholipids, and Molar Ratio

Phospholipids, triglycerides, and total cholesterol levels in the artery tissue are displayed in Table 5, which has implications for cardiovascular disease risk. Molar ratio analysis was performed in order to shed light on the structure and operation of cell membranes as well as the equilibrium among various lipid components in the body (Table 5). As anticipated, the molar ratio, phospholipids, TC, and TG of the induced-obese group (G2) were significantly higher (*p* < 0.05) than the G1 (control group), an outcome that was dramatically counteracted when supplement I (HFD−WKBE) was given to G3. This significant reduction (*p* < 0.05) in the artery tissue lipid profile was amplified by co-treating with either PEE alone or with CrPi_3_, where TG and phospholipid levels did not differ significantly (*p* < 0.05) among rats in G5 from those of the G1 control group.

**Figure 6:**
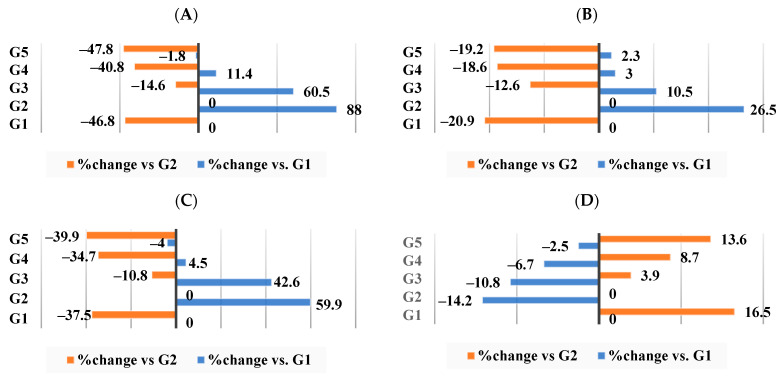
Percentage of changes in artery lipid profile among all groups. (**A**) Total cholesterol. (**B**) Triglyceride. (**C**) Phospholipids. (**D**) Molar ratio.

In comparison to the obese group (G2), WKBE displayed a percentage change in the artery TC (−14.6%), TG (−12.6%), phospholipids (−10.8%), and molar ratio (3.9%). Conversely, G3-supplement II, which contained PEE, displayed percentage changes in the artery’s TC (−40.8%) (Figure 6A), TG (−18.6%) (Figure 6B), phospholipids (−34.7%) (Figure 6C), and molar ratio (8.7%) (Figure 6D). The addition of CrPi_3_ produced the best results, with percentage changes in these parameters being −47.8% for the artery TC, −19.2% for the artery TG, −39.9% for the artery phospholipids, and 13.6% for the molar ratio (Figure 6A–D).

### 3.6. Effect of Different Nutraceutical Supplements on Total Liver Tissue Lipids, Total Cholesterol, and Triglyceride Levels

As an indication for dyslipidemia, we evaluated the hepatic levels of total lipids, total cholesterol, and triglyceride levels. Table 6 demonstrates that G2 depicted a significant increase (*p* < 0.05) in total hepatic lipids, cholesterol, and triglycerides compared to the control group (G1). At the same time, G3, G4, and G5 showed a significant reduction (*p* < 0.05) in these values. G5 showed a hepatic triglyceride value that was insignificantly different (*p* > 0.05) from G1 (control group).

**Figure 7:**
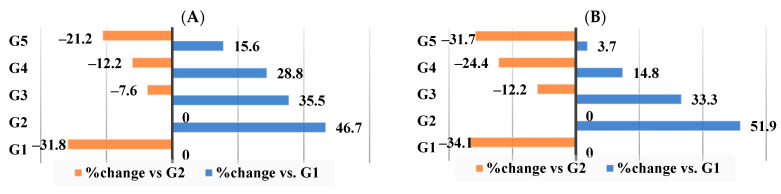

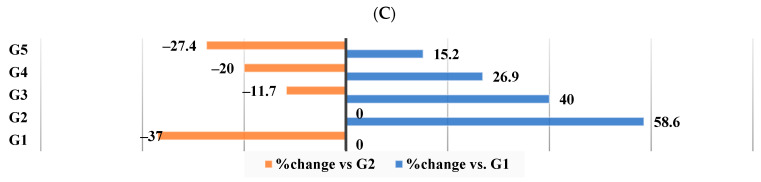
Percentage of changes in liver lipid profiles. (**A**) Total lipids. (**B**) Total cholesterol. (**C**) Triglyceride.

As per Figure 7A–C, the most significant outcomes were seen in supplement III, which was given to G5. The percentage changes in total liver lipids were −21.2%, total cholesterol −31.7%, and triglycerides −27.4%. In contrast, G4 demonstrated changes of −12.2%, −24.4%, and −20%, respectively, and G3 demonstrated changes of 7.6%, −12.2%, and −11.7%, respectively.

### 3.7. Effect of Different Nutraceutical Supplements on Liver Enzymes in Obese Rats

The hepatoprotective effect of the supplements among the three experimental groups G3, G4, and G5 can be assessed using liver enzymes, which are biomarkers for hepatotoxicity. The liver enzyme levels were significantly reduced (*p* < 0.05) by supplements I and II, which were fed to groups G3 and G4, respectively, compared to the G2 group. But as Table 7 demonstrates, co-administering CrPi_3_ along with HFD–WKBE–PEE allowed the liver enzyme levels in the G5 group to return to normal, with rats expressing liver enzyme levels that were not statistically different (*p* > 0.05) from the control group (G1).

**Figure 8:**
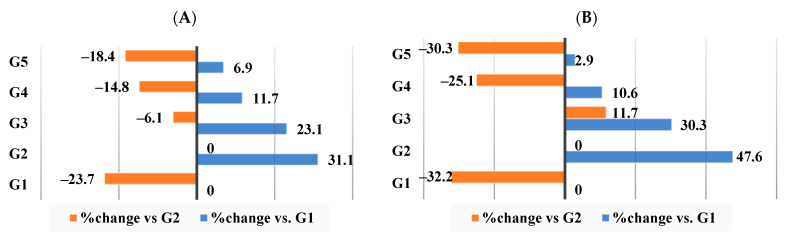

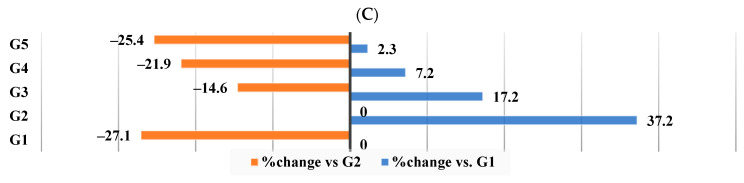
Percentage of changes in levels of liver enzymes. (**A**) AST level. (**B**) ALT level. (**C**) ALP level.

The alterations depicted in Figure 8A–C demonstrated that, in comparison to the control group (G1) fed a basic diet, HFD increased the liver enzyme levels in G2 by 13.1% for AST, 47.6% for ALT, and 37.2% for ALP. In comparison to G2, WKBE revealed changes in the AST, ALT, and ALP of (−6.1%, −11.7%, and −14.6%, respectively). In addition, changes were observed in supplements II and III for AST, ALT, and ALP of −14.8%, 25.1%, and −21.9%, and −18.4%, −25.4%, and −30.3%, respectively.

### 3.8. Effect of Different Nutraceutical Supplements on Kidney Functions and Glucose Levels

The mean values of urea, creatinine, uric acid, and glucose in the serum of the obesity-induced group (G2) were significantly increased (*p* < 0.05) compared with those of the negative control group (G1) as a result of induced obesity, shown in Table 8. Administering supplement I, II, or III to these obese rats significantly decreased (*p* < 0.05) urea, creatinine, uric acid, and glucose compared with those of G2. G5 showed the least significant values as compared to G2 and showed a normalization in the glucose level as compared to the control group (G1).

**Figure 9:**
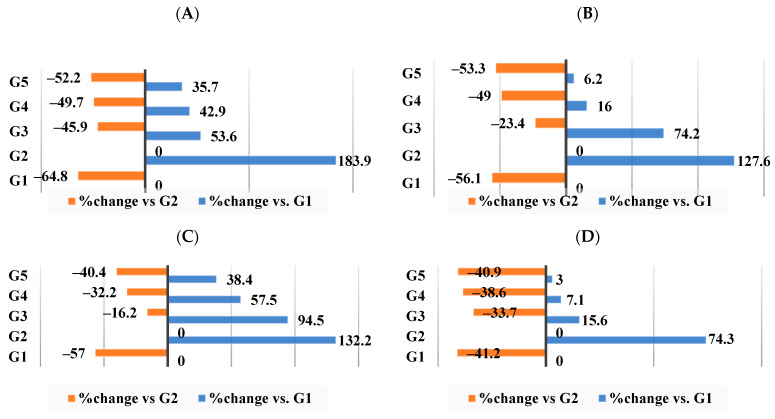
Percentage of changes in kidney function biomarkers. (**A**) Blood Urea Nitrogen (BUN) level. (**B**) Creatinine level. (**C**) Uric acid level. (**D**) Glucose level.

As seen in Figure 9A–D, the G2 group that received the HFD had changes in kidney function biomarkers, such as elevated blood urea nitrogen (183.9%), creatinine (1276.6%), uric acid (123.2%), and glucose (74.3%) in comparison to the G1 control group. Supplement I (HFD–WKBE) administration reduced this rise and caused these biomarkers to shift by −45.9%, −23.4%, −16.2%, and −33.7%, respectively. When rats were fed supplements II (HFD–WKB–PEE) and III (HFD–WKBE–PEE–CrPi_3_), further attenuation was achieved in blood urea nitrogen changed by −49.7% and −52.2% in groups G4 and G5, respectively.

### 3.9. Effect of Different Nutraceutical Supplements on Concentration and Profile of the Short-Chain Fatty Acids (SCFAs) and Colonic pH

**Figure 10:**
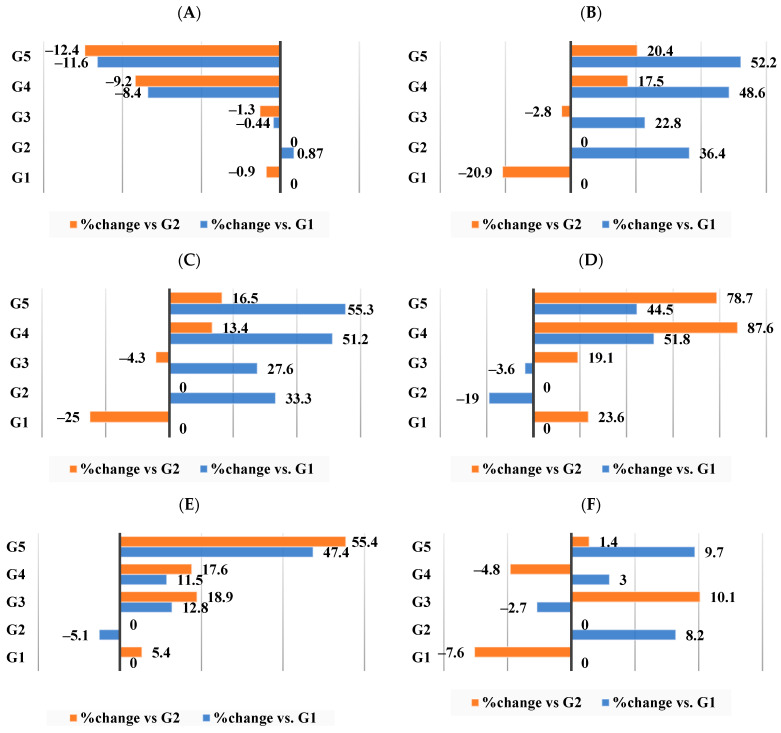
Percentage of changes in colonic pH, SCFA concentration, and profile among all groups. (**A**) Colonic pH. (**B**) SCFA concentration. (**C**) Acetic acid. (**D**) Propionic acid. (**E**) Isobutyric acid. (**F**) Butyric acid.

Table 9 presents our data, which indicate that compared to G1, the colonic pH for the obesity-induced group (G2) showed a minor increase of 0.87% (Figure 10A) while that of G3 showed a minor decrease of −0.44% change (Figure 10A)—a result that improved for both G4 and G5, indicating a significantly lower (*p* < 0.05) colonic pH when compared to the obese group (G2) with a change of −9.2% and −12.4%, respectively (Figure 10A). In accordance with these findings, the concentration of total SCFAs in G3 was slightly lower than in G2 with a change of −2.8% (Figure 10B), whereas G4 had a significant increase (*p* < 0.05) with a percentage of change of 17.5%, and G5 had the largest significant increase (*p* < 0.05) with a percentage of change 20.4% (Figure 10B). Both acetic acid and propionic acid were the dominant SCFAs in the colonic content of all the groups. Their values were significantly increased (*p* < 0.05) in the colonic content of the rats in G5 followed by G4 then G3. Isobutyric acid and butyric acid made up a smaller percentage of SCFAs than acetic acid and propionic acid, but they also demonstrated a comparable and significant increase (*p* < 0.05) in G4 and G5 when compared to G2.

Figure 10A–F shows the percentage of changes in the acids representing the SCFA profile. The highest percentages of change in acetic acid, propionic acid, and isobutyric acid concentrations were achieved among G5 (16.5%, 78.7%, and 55.4%, respectively). Meanwhile, as per Figure 10F, G3 showed the highest percentage of change for butyric acid (10.1%).

### 3.10. Effect of Different Nutraceutical Supplements on Total Fecal Lipids, Cholesterol, and Triglycerides

The total fecal lipids, cholesterol, and triglycerides in the G2 (induced-obese) group were significantly lower (*p* < 0.05) than those in the G1 control group, as shown in Table 10. As expected, G3 had considerably higher fecal lipid content, including total lipids, cholesterol, and triglycerides, followed by G4 and finally G5, which had the highest levels.

**Figure 11:**
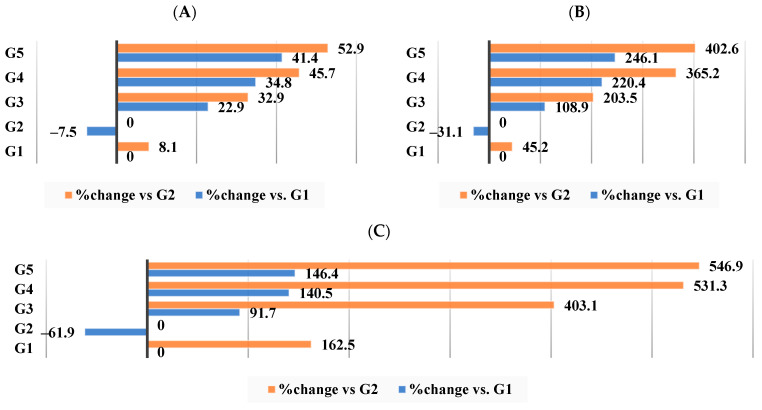
Percentage of changes in fecal lipid content among all groups. (**A**) Total lipids in feces. (**B**) Total cholesterol in feces. (**C**) Triglycerides in feces.

Figure 11A–C demonstrates that when HFD−WKBE was administered to G3, the total lipid concentration changed by 32.9% in comparison to the obesity-induced group (G2). In contrast, when PEE was added (G4) or CrPi_3_ was added (G5), the percentage change was 45.7% and 52.9%, respectively (Figure 11A). Moreover, there was a change of 203.5% (Figure 11B) and 403.1% (Figure 11C) in total triglycerides and cholesterol in G3. The percentage change in triglycerides and total cholesterol after co-administration of PEE was 531.3% (Figure 11C) and 365.2% (Figure 10B), respectively. Ultimately, the largest change was seen in G5, where triglycerides were 546.9% (Figure 11C) and total cholesterol was 402.6% (Figure 11B).

## 4. Discussion

Obesity has been linked to a range of metabolic conditions, such as insulin resistance, type 2 diabetes, dyslipidemia, hepatic steatosis, and cardiovascular diseases [50]. Dietary interventions are becoming a key tactic in the fight against obesity and the problems that come with it. In order to reverse obesity and its related issues, our study examined the benefits of combining dietary supplements with anti-obesity properties. Our findings are quite promising because we were able to show that the combination of WKB, PEE, and CrPi_3_ reversed obesity, had some positive benefits, and occasionally even outperformed the use of each supplement alone. Our study is unique in that it examines the impact of 18 weeks of dietary supplementation with various supplements on all aspects of obesity, including body weight gain, food intake, relative organ weight, serum lipid profile, and protective effects on the gut microbiota, liver, and kidneys.

It is critical to identify and measure the bioactive components of natural products in order to better understand their pharmacological effects and efficacy. Initially, we evaluated the α-amylase inhibitory activity of the utilized white kidney bean extract against porcine pancreatic amylase. The results showed that it was 3000 U/g, which falls within the previously reported range of 1840–5777 U/g [51]. Strong antioxidants, flavonoids, and polyphenols can effectively combat free radicals that cause oxidative stress [52]. As was previously mentioned, the amount of antioxidant (flavonoids and nonflavonoids) contents found in propolis is significantly influenced by the pedoclimatic features of the collection region, the production conditions, and the harvesting period. This contributes to the explanation of the notable differences found between the assessed samples and additional propolis samples from different locations [53]. Our findings show that the antioxidant phenolic and flavonoid contents of the propolis ethanolic extract have an abundant profile. These values are In close agreement with the data reported for a propolis sample that was harvested from the same area [54]. 

In order to accomplish our main goal, we fed a high-fat diet (HFD) to one group of rats in order to induce obesity. We then used that group exclusively as the positive control group so we could confirm that the HFD caused obesity and its consequences, such as an increase in the relative weight of organs, and abdominal fat, which was associated with complications such as hyperlipidemia, hyperglycemia, and impaired functionality of the kidneys and liver. We examined the anti-obesity effects of supplement I (HFD–WKBE), supplement II (HFD–WKBE–PEE), supplement III (HFD–WKBE–PEE–CrPi_3_), and the basic diet after 18 weeks of feeding the five groups of rats. Supplement III had the greatest effects on reducing body weight increase, food consumption, and food utilization rate, according to our results. This can be explained by the impact of WKBE, which has been demonstrated to play a major part in the treatment of non-alcoholic fatty liver, obesity, and type 2 diabetes [55,56]. According to reports, WKBE has pharmacological effects that lower body weight, appetite, hyperlipidemia, and gut microbiota regulation. The primary reason behind these consequences is the inhibition of α-amylase (α-AI), which reduces the rate at which complex carbohydrates undergo digestion and, in turn, the rate at which the stomach empties [57,58,59,60]. A cholecystokinin-mediated mechanism has been proposed as the reason for the decrease in the intake of food [61]. The main components of propolis are thought to be flavonoids, which can also prevent fragility, lipid peroxidation, platelet aggregation, and capillary permeability [62]. On the other hand, CrPi_3_ is a commonly used dietary supplement that has been researched for possible effects on controlling appetite and weight [63,64].

The HFD group’s induced obesity resulted in a notable increase in abdominal fat and relative weight of some organs, which was significantly attenuated along with the reduction in body weight gain effect. The administration of supplements II and III had a positive effect on the relative weight of organs and abdominal fat. While WKBE alone in supplement I did not normalize these values relative to the control group, PEE supplementation resulted in significant reductions in abdominal fat, preservation of some organs, and normalization of the relative weights of the spleen and kidneys—an effect that has been previously documented [65,66]. Although the results were not significantly changed by adding CrPi_3_ to PEE, supplement III showed the highest recovery. Previous studies on the CrPi_3_ relative weight of organs yielded mixed results; we found an increase in relative organ weights compared to the control group, whereas some studies reported insignificant changes in relative organ weights compared to the normal group [63]. However, our results are in line with other studies that found a decrease in the absolute and relative weights of the organs when compared to the obese group [67].

A significant part of the pathophysiology of lipid metabolism in obesity is determined by lipid profile, accumulation, and excretion. We assessed the serum and artery lipid profiles to look into how the three nutraceutical supplements affected other characteristics of obesity, such as hyperlipidemia and the risk of cardiovascular disease. Atherosclerosis usually begins with low-density lipoprotein (LDL) oxidation, which sets off inflammatory and immunological reactions that eventually result in the formation of atherosclerotic plaque and cardiovascular disease [68,69]. In addition to keeping LDL from oxidizing, HDL scavenges excess LDL-C from peripheral tissues and transports it to the liver for metabolism and excretion. Moreover, HDL may lower or even reverse the effects of oxidized LDL in the walls of arteries [70]. Thus, elevated serum concentrations of TC, TG, and LDL-C are linked to a higher risk of hyperlipidemia, atherosclerosis, and other cardiovascular conditions, while higher HDL-C levels are linked to a lower risk of these illnesses. The serum and arterial lipid profile were significantly improved by co-treatment with PEE and CrPi_3_ in supplement III. These improvements included low TG, TC, LDL-C, and VLDL-C as well as high HDL-C levels in serum, which were consistent with other studies [71,72,73]. As a result, the artery tissue lipid profile, coronary risk ratio (CRR), atherogenic index (AI), and calculated HDL-C/LDL-C ratio were all significantly lower. In comparison to the control group, chromium picolinate not only significantly lowered the values of the artery tissue lipid profile but also normalized them to a non-significant level [74].

The level of fat absorbed from the intestine can be measured by looking at fecal lipids. The obese group had lower fecal lipid and cholesterol levels than the control group. Fecal lipid levels were highest in supplement III, indicating a more significant impact of propolis extract and CrPi_3_ on intestinal fat absorption. This implies that propolis may help reduce weight by encouraging bowel movements and preventing the absorption of fat [75]. The precise workings of this mechanism are still unknown, though, as there has been little research on the effect of CrPi_3_ on fecal content.

Previous theories attributed the beneficial effects of kidney beans on lowering rats’ total serum cholesterol, providing a protective effect against hyperlipidemia and cardiovascular diseases, to their high dietary fiber content, soluble saponins, polyphenolics, tannins, and vegetable proteins [76,77]. There have been alternative theories suggesting that the in vitro cholesterol-lowering effect of WKBE is caused by its protein hydrolysates [77,78].

Research on rat models of diabetes or dyslipidemia has documented the hypolipidemic and lipid peroxidation effects of PEE [79,80,81]. Propolis’s ability to inhibit the synthesis of cholesterol by lowering the level of β-Hydroxy β-methylglutaryl-CoA (HMG-CoA) reductase protein in the liver has been identified as its mechanism of action [82]. PEE polyphenols and flavonoids have been linked to a lower risk of cardiovascular disease as a result of mediating the rise in plasma HDL-C levels [83].

There are two potential explanations for how PEE works to lower LDL lipid peroxidation. The first is by activating the Nuclear Factor Erythroid 2-related Transcription Factor 2 (NRF2), which leads to an increase in the expression of antioxidant enzymes such as heme oxygenase-1, phase II detoxification, and glutathione (GSH) metabolism [84]. The second mechanism might involve suppressing pro-inflammatory genes by neutralizing oxidative species and obstructing signaling pathways leading to nuclear transcription factor-kappa B (NF-κB) activation [85].

Although the exact mechanism through which CrPi_3_ reduces the lipid profile is still unknown, studies have shown that it is related to the way that proteins involved in cholesterol homeostasis, synthesis, uptake, and efflux within cells respond to CrPi_3_ [74,86]. The downregulation of ATP-binding cassette transporter-A1 (ABCA1) is thought to be the reason for CrPi_3_’s attenuation effect on lipids, which is consistent with SREBP-mediated transcriptional repression of the ABCA1 gene. Membrane-bound transcription factors known as sterol regulatory element-binding proteins (SREBPs) have been shown to upregulate numerous genes involved in cholesterol biosynthesis, including HMG-CoA (3-hydroxy-3-methylglutaryl-CoA) reductase. Concurrently, SREBPs inhibit ABCA1 expression. ABCA1 is involved in the reverse cholesterol transport pathway and the cellular efflux of extra cholesterol back to the liver for excretion, both of which are critical for preserving cellular cholesterol homeostasis [87,88].

A diet high in fat increases free fatty acid levels in blood and overloads the liver with fat and cholesterol, all of which can cause hepatotoxicity, dyslipidemia, excess body fat accumulation, and cardiovascular diseases. As a result, long-chain fatty acid peroxidation replaces the regular mitochondrial lipid oxidation process, increasing lipotoxicity and yielding a remarkable amount of malonaldehyde, a byproduct of lipid peroxidation and a marker of reactive oxygen species (ROS) [89,90]. When compared to the obese rats fed HFD alone, supplement III treatment dramatically reduced the levels of malondialdehyde, a marker of lipid peroxidation and an indicator of oxidative stress. Previous studies have shown that this action is caused by flavonoids, specifically flavanols like quercetin, which are present in both PEE and WKBE. By opposing the effects of reactive oxygen and nitrogen species, which limit lipid peroxidation, flavonoids serve as antioxidants [90]. This mechanism impacts not just the cardiovascular system but also the liver, which is the organ responsible for lipid and lipoprotein metabolism. In addition, when compared to the control group, our results showed that treatment with CrPi_3_ caused the levels of MDA oxidative stress indicator to return to normal. Our results are in line with past studies that found that rats fed a high-fat diet and given chromium supplements had lower MDA levels. This shows that obesity-related oxidative stress, which was initially brought on by an imbalance between pro-oxidants and antioxidants, may be lowered by chromium. It is believed that Cr accomplishes this function by boosting insulin and antioxidant enzyme activity, which lowers lipid peroxidation [91,92].

The liver is crucial for maintaining glucose and lipid homeostasis because it produces, stores, and exports lipids and lipoproteins, plays a significant role in lipid metabolism, and imports serum free fatty acids. Concentrations of liver enzymes such as alkaline phosphatase (ALP), alanine aminotransferase (ALT), and aspartate aminotransferase (AST) are used as screening markers for hepatotoxicity. These enzymes are specific to the liver and provide valuable insights into its operation; they increase when the liver is damaged or malfunctioning.

Hepatic steatosis is caused by an imbalance between lipogenesis/influx and lipolysis/export, which builds up triglycerides in the liver. Thus, another crucial indicator of the liver’s state of function is its lipid content. Our findings showed that supplement III stopped fat from building up and stopped steatosis from developing in the liver as a result of a high-fat diet. As a result, total lipids, total cholesterol, and total triglycerides decreased significantly or returned to normal, as did the hepatic enzymes (ALT, AST, and ALP). Although PEE and CrPi_3_ had previously shown this effect [23,93,94], in our study they both functioned in conjunction to prevent or manage obesity-induced liver damage. There is growing evidence that legumes such as WKBE are an excellent source of protein, dietary fiber, and polyphenols that also naturally protect the liver through a variety of mechanisms [58,94]. It is thought that the extract from white kidney beans protects against hepatic steatosis by improving the condition of the liver and biliary duct, where dietary fiber plays a significant role in controlling metabolites and the gut microbiota [60,95]. Chromium’s importance as a hepatoprotective agent and enhancer of hepatic function has been highlighted by previous research that demonstrated significantly reversed hepatocyte injury and hepatic triglyceride buildup in rats fed a high-fat diet [64,92]. Propolis and chromium picolinate have been shown to have effects on liver metabolism; these findings may be explained by molecular mechanisms involving peroxisome proliferator-activated receptors (PPARs) [93]. Propolis may contain ligands for the peroxisome proliferator-activated receptor gamma protein (PPAR-γ). These ligands, being PPAR-γ agonists, have the ability to decrease the amount of fat buildup in the rats’ livers following a high-fat diet. Propolis enhances lipolysis overall by lowering the activity of enzymes involved in cholesterol production, decreasing the activity of hormone-sensitive lipase, and increasing the activity of the hepatic enzyme glucokinase [66,88]. Concurrently, chromium picolinate is associated with hepatic upregulation of PPARα. The regulation of fatty acid metabolism, oxidative catabolism, inflammatory mechanisms, and energy homeostasis is linked to the activation of the hepatic lipid catabolic transcription factor, PPARα [96,97,98]. Furthermore, PPARα contributes to the amelioration of insulin resistance by reducing the dysregulation of intracellular insulin signaling cascade and protein expression. Subsequently, this elevates the beta-oxidation of fatty acids in the liver, which is the major process by which fatty acids are oxidized to generate energy [99].

Elevated kidney levels of glucose, urea, uric acid, and creatinine are important markers of issues related to obesity. Our data showed that following the 18-week HFD, these parameters were significantly increased in the induced-obese rats more than the control group—an outcome that was substantially mitigated by supplement III and reversed by giving both supplements I and II. Our results are supported by earlier studies on the effects of a WKBE-fed diet on rats’ kidney functions and blood glucose levels. These studies also observed a significant reduction in the elevated blood glucose level brought on by a high-fat diet in rats, as well as in the area under the curve of the time course of glycaemia and the postprandial glucose level [58,59,100]. This is in accordance with other PEE-based research that showed markedly elevated serum biochemical markers for renal function and insulin resistance [66]. Propolis has been related to improvements in albumin glomerular leakage, decreased microalbuminuria, and elevated hepatorenal glutathione peroxidase levels in relation to kidney function [81,93].

Activation of the inhibitor NF-κB kinase (IKK) is triggered by obesity, hyperglycemia, and elevated free fatty acids. This, in turn, causes the release of NF-κB and the nuclear factor of kappa light polypeptide gene enhancer in B-cells (IκBα). Insulin resistance is the result of prolonged NF-κB activation brought on by the liver’s overexpression of IKK [84]. Several theories have been proposed to explain the hypoglycemic effect of propolis, such as the protective action on β-cells, which could improve the production of insulin, boost cellular sensitivity responses to insulin, which may increase glucose utilization in peripheral tissues, and inhibit the enzyme α-glucosidase, which may lessen intestinal glucose absorption [101]. In consistent with previous studies, the addition of chromium picolinate in our investigation produced further improvements, especially for the glucose level, which was lowered to almost normal levels with no significant change from the rats in the control group. According to reports, CrPi_3_ improves β-cell sensitivity, insulin receptor number, and insulin internalization to reduce insulin resistance [63]. Like PEE, CrPi_3_ modifies the renal NF-κB pathway brought on by obesity-induced insulin resistance and reactive oxygen species production. In the end, Iκ-Bα levels are partially restored by CrPi_3_ [102]. Another pathway is the intracellular protein that interacts with the insulin receptor, which is widely distributed in the kidneys, liver, spleen, and intestine. This peptide increases tyrosine kinase activation and insulin receptor activity [103].

Carbohydrates and partially and nondigestible polysaccharides are fermented by the gut microbiota, into a significant number of fatty acid molecules called saturated fatty acids (SCFAs) which are good for gut health [104]. There is a strong correlation between a lower colonic pH and an elevated intestinal SCFA concentration. Butyric, propionic, and acetic acids are the three main SCFAs that comprise the SCFA profile: acetic acid accounts for more than 95% of the total [104]. According to certain reports, the metabolism of fat and glucose involves the involvement of all three acids. Acetic and propionic acids help regulate body weight and insulin sensitivity [104]. Butyric acid not only keeps colon cancer away, but it also keeps the environment inside the intestines stable. The most notable improvements were seen in supplement III in the colonic pH, SCFA profile, and SCFA concentration (propionic acid and acetic acid were the predominant SCFAs, with isobutyric and butyric acid accounting for smaller proportions). This outcome can be explained by the extract from white kidney beans’ high polyphenol content, which improved the gut microbiota’s composition and encouraged the rat colon’s production of SCFAs while reducing inflammation [104].

Furthermore, it was found that providing WKBE significantly increased the concentration of propionic acid. Propionic acid inhibits HMG-CoA activity in the liver and lowers serum lipid levels, both of which are advantageous for metabolic health in general and lipid-lowering in particular. Previous studies have demonstrated beneficial effects such as a stabilization of the gut microbiota and an increase in beneficial bacteria; PEE and CrPi_3_ have been shown to further enhance these effects [105,106,107]. This result contradicted the findings of some other studies, but it confirmed our theory that propolis and chromium-containing dietary supplements affected intestinal microbial activity [108]. Increased SCFA synthesis has been shown to impact lipogenesis and the secretion of peptide YY (PYY), which is released postprandially and can therefore suppress appetite and food intake, preventing body weight gain [109].

## 5. Conclusions

To summarize, in the present study we aimed to determine the effectiveness of combining the bioactive substances in the three nutraceutical supplements containing white kidney bean extract (WKBE), propolis ethanolic extract (PEE), and CrPi_3_ on an obesity-induced rat model and compare their anti-obesity effects with normal and obese rats. Comparable effects included nutritional parameters, biochemical parameters, and biomarkers of cardiovascular disease, liver function, kidney function, and gut health. Using Sprague-Dawley rats fed a 45% high-fat diet we demonstrated a promising outcome of combining the three nutraceutical supplements (WKBE, PEE, and CrPi_3_) in the management of obesity and its complications. Throughout the entire study, the positive control rats were kept solely as a reference to show the effect of the high-fat diet and confirm the reverting or normalizing effect of the investigational diets. Overall, the results indicated a synergetic effect in reducing weight gain, food utilization rate, abdominal fat, serum lipids, arterial and hepatic lipids, risk of cardiovascular disease, and blood glucose level, in addition to improving renal function and gut microbiota. WKBE primarily exerted its effect through its α-amylase inhibitor action and high dietary fiber content which led to a reduction in the complex carbohydrate digestion rate, gastric emptying rate, and intake of food. PEE helped reduce weight by encouraging bowel movements and preventing the absorption of fat due to its high dietary fiber content, soluble saponins, polyphenolics, tannins, and vegetable proteins. Flavonoids and polyphenols found in WKBE and PEE worked together to reduce hyperlipidemia, risk of cardiovascular disease, and lipid peroxidation, and provided a hepatoprotective effect. On the other hand, CrPi_3_—a dietary supplement that is frequently used and has effects on appetite and weight control—acted as a catalyst to the WKBE and PEE, which resulted in a further reduction or even normalization of certain parameters. Furthermore, we found no adverse effects of combining the three nutraceuticals in any organ or on the general health of rats over the 14-week duration of the study or doses used in the three investigational diets. Pre-clinical research frequently uses the rat model, but it is important to remember that findings from these studies cannot be applied directly to humans without additional clinical research. Therefore, in order to translate the knowledge gained from this study to clinical intervention, our future research will aim to formulate a nutraceutical supplement in the dosage form of tablets, sachets, and capsules, while following the FDA daily recommendation, with the goal of investigating it in a clinical trial setting.

## Figures and Tables

**Table 1:** Experimental and basic diet compositions (g/1000 g diet).

Groups	G1 (−ve) Control Group	G2 (+ve) Control Group	G3 Supplement I	G4 Supplement II	G5 Supplement III
Corn Starch	397.486	331.986	326.986	324.486	324.485
Casein (≥85% protein)	200	200	200	200	200
Dextrinized corn starch	132	70	70	70	70
Sucrose	100	50	50	50	50
Soybean oil	70	70	70	70	70
Lard	-----	177.50	177.50	177.50	177.50
Non-nutritive cellulose	50	50	50	50	50
Mineral mix	35	35	35	35	35
Vitamin mix	10	10	10	10	10
L-Cysteine	3	3	3	3	3
Choline bitartrates (41% choline)	2.50	2.50	2.50	2.50	2.50
Tert-Butylhydroquinone	0.014	0.014	0.014	0.014	0.014
White kidney bean (WKB) extract (3000 unit/g α-AI)	-----	-----	5	5	5
Propolis ethanolic extract (PEE)	-----	-----	-----	2.50	2.50
Chromium picolinate (Chromium Eq. 120 ug)	-----	-----	-----	-----	0.001
Total	1000	1000	1000	1000	1000

**Table 2:** Effects of different nutraceutical supplements on initial weight, final weight, body weight gain, food consumption, and food utilization rate (%) in HFD-induced obese rats.

Groups	G1	G2	G3	G4	G5
Initial weight (g)	370.5 ± 5.9 ^b^	434.4 ± 4.9 ^a^	434.9 ± 4.9 ^a^	433.6 ± 4.9 ^a^	434.3 ± 4.9 ^a^
Final weight (g)	434.9 ± 6.3 ^c^	539.2 ± 6.1 ^a^	511.9 ± 6.2 ^b^	500.2 ± 5.7 ^b^	494.1 ± 5.6 ^b^
Body weight gain (g)	64.4 ± 0.7 ^c^	104.9 ± 1.2 ^a^	76.0 ± 0.9 ^b^	66.6 ± 0.8 ^c^	59.8 ± 0.7 ^d^
Food consumption (g)	380.6 ± 4.3 ^a^	366.2 ± 4.2 ^b^	357.1 ± 4.1 ^b^	343.0 ± 3.9 ^c^	330.5 ± 3.8 ^d^
Food utilization rate (%) *	16.9 ± 0.004 ^e^	28.5 ± 0.003 ^a^	20.3 ± 0.004 ^b^	19.4 ± 0.003 ^c^	18.1 ± 0.003 ^d^

* Food utilization rate (%) = (body weight gain/food consumption) × 100. Results are presented as the mean ± SE, *n* = 9. Differences were considered statistically significant at *p* < 0.05. Superscript letters (a, b, c, d, e) denote statistical differences between groups.

**Table 3:** Effect of different nutraceutical supplements on the relative weight of organs and tissues in obese rats.

Groups	G1	G2	G3	G4	G5
Liver	4.7 ± 0.05 ^d^	6.0 ± 40.07 ^a^	5.4 ± 0.06 ^b^	5.2 ± 0.06 ^c^	5.1 ± 0.06 ^c^
Kidney	1.15 ± 0.01 ^c^	1.3 ± 0.02 ^a^	1.21 ± 0.01 ^b^	1.18 ± 0.01 ^c^	1.16 ± 0.01 ^c^
Spleen	0.71 ± 0.01 ^c^	0.82 ± 0.01 ^a^	0.78 ± 0.01 ^b^	0.73 ± 0.01 ^c^	0.72 ± 0.01 ^c^
Heart	0.44 ± 0.005 ^e^	0.55 ± 0.007 ^a^	0.52 ± 0.007 ^b^	0.48 ± 0.005 ^c^	0.46 ± 0.005 ^d^
Aorta	0.06 ± 0.001 ^d^	0.13 ± 0.001 ^a^	0.09 ± 0.001 ^b^	0.071 ± 0.001 ^c^	0.07 ± 0.001 ^c^
Intra-abdominal fat	5.1 ± 0.06 ^d^	5.9 ± 0.07 ^a^	5.7 ± 0.07 ^b^	5.5 ± 0.06 ^c^	5.4 ± 0.06 ^c^

Results are presented as the mean ± SE, *n* = 9. Differences were considered statistically significant at *p* < 0.05. Superscript letters (a, b, c, d, e) denote statistical differences between groups.

**Table 4:** Effect of different nutraceutical supplements on lipid profiles.

Groups	G1	G2	G3	G4	G5
TG (mmol/L)	0.62 ± 0.002 ^e^	1.9 ± 0.02 ^a^	1.69 ± 0.02 ^b^	1.37 ± 0.02 ^c^	1.22 ± 0.01 ^d^
Phospholipids (mmol/L)	1.61 ± 0.02 ^b^	1.72 ± 0.02 ^a^	1.31 ± 0.02 ^c^	1.27 ± 0.01 ^c d^	1.22 ± 0.01 ^d^
MDA (umol/L)	2.32 ± 0.03 ^c^	4.39 ± 0.05 ^a^	3.44 ± 0.04 ^b^	2.46 ± 0.03 ^c d^	2.39 ± 0.03 ^d^
TC (mmol/L)	2.41 ± 0.03 ^e^	3.02 ± 0.03 ^a^	2.9 ± 0.03 ^b^	2.72 ± 0.03 ^c^	2.63 ± 0.03 ^d^
HDL-C (mmol/L)	1.93 ± 0.02 ^a^	1.61 ± 0.02 ^d^	1.69 ± 0.02 ^c^	1.78 ± 0.02 ^b^	1.82 ± 0.02 ^b^
LDL-C (mmol/L)	0.36 ± 0.004 ^e^	1.03 ± 0.01 ^a^	0.87 ± 0.01 ^b^	0.65 ± 0.007 ^c^	0.56 ± 0.007 ^d^
VLDL-C (mmol/L)	0.12 ± 0.001 ^e^	0.38 ± 0.005 ^a^	0.34 ± 0.004 ^b^	0.28 ± 0.003 ^c^	0.25 ± 0.003 ^d^
Atherogenic index (AI)	0.25 ± 0.003 ^e^	0.87 ± 0.01 ^a^	0.71 ± 0.008 ^b^	0.52 ± 0.007 ^c^	0.44 ± 0.005 ^d^
Coronary risk ratio	1.25 ± 0.01 ^d^	1.86 ± 0.02 ^a^	1.70 ± 0.02 ^b^	1.51 ± 0.02 ^c^	1.13 ± 1.01 ^e^
HDL-C/LDL-C	5.37 ± 0.06 ^a^	1.56 ± 0.02 ^e^	1.94 ± 0.02 ^d^	2.69 ± 0.03 ^c^	3.26 ± 0.04 ^b^
LDL-C/HDL-C	0.19 ± 0.003 ^e^	0.64 ± 0.007 ^a^	0.51 ± 0.007 ^b^	0.37 ± 0.004 ^c^	0.31 ± 0.004 ^d^

Results are presented as the mean ± SE, *n* = 9. Differences were considered statistically significant at *p* < 0.05. Superscript letters (a, b, c, d, e) denote statistical differences among groups.

**Table 5:** Effect of different nutraceutical supplements on artery tissue (total cholesterol, triglyceride, phospholipids, and molar ratio) in obese rats (mg/g tissue).

Groups	G1	G2	G3	G4	G5
TC (mg/g)	1.67 ± 0.02 ^d^	3.14 ± 0.04 ^a^	2.68 ± 0.03 ^b^	1.86 ± 0.02 ^c^	1.64 ± 0.02 ^d^
TG (mg/g)	25.33 ± 0.29 ^c^	32.04 ± 0.36 ^a^	27.99 ± 0.32 ^b^	26.09 ± 0.3 ^c^	25.9 ± 0.29 ^c^
Phospholipids (mg/g)	2.02 ± 0.02 ^d^	3.23 ± 0.04 ^a^	2.88 ± 0.03 ^b^	2.11 ± 0.02 ^c^	1.94 ± 0.02 ^d^
Molar ratio (Phospholipid/TC)	1.20 ± 0.01 ^a^	1.03 ± 0.01 ^d^	1.07 ± 1.01 ^c^	1.12 ± 0.01 ^b^	1.17 ± 1.01 ^a^

Results are presented as the mean ± SE, *n* = 9. Differences were considered statistically significant at *p* < 0.05. Superscript letters (a, b, c, d) denote statistical differences between groups.

**Table 6:** Effect of different nutraceutical supplements on total liver tissue lipids, total cholesterol, and triglycerides (mg/g wet liver).

Groups	G1	G2	G3	G4	G5
Total lipids	44.8 ± 0.51 ^b^	65.7 ± 0.75 ^a^	60.7 ± 0.69 ^b^	57.7 ± 0.66 ^c b^	51.8 ± 0.59 ^a d^
Total cholesterol	2.7 ± 0.03 ^e^	4.1 ± 0.05 ^a^	3.6 ± 0.04 ^b^	3.1 ± 0.03 ^c^	2.8 ± 0.03 ^d^
Triglyceride	14.5 ± 0.17 ^d^	23.0 ± 0.26 ^a^	20.3 ± 0.23 ^b^	18.4 ± 0.21 ^c^	16.7 ± 0.19 ^d^

Results are presented as the mean ± SE, *n* = 9. Differences were considered statistically significant at *p* < 0.05. Superscript letters (a, b, c, d, e) denote statistical differences among groups.

**Table 7:** Effect of different nutraceutical supplements on liver function enzymes (mg/g wet liver).

Groups	G1	G2	G3	G4	G5
AST activity	57.9 ± 0.66 ^e^	75.9 ± 0.86 ^a^	71.3 ± 0.81 ^b^	64.7 ± 0.74 ^c^	61.9 ± 0.7 ^d^
ALT activity	20.8 ± 0.24 ^d^	30.7 ± 0.35 ^a^	27.1 ± 0.31 ^b^	23.0 ± 0.26 ^c^	21.4 ± 0.24 ^d^
ALP activity	77.9 ± 0.89 ^d^	106.9 ± 1.2 ^a^	91.3 ± 1.04 ^b^	83.5 ± 0.95 ^c^	79.7 ± 0.91 ^d^

Results are presented as the mean ± SE, *n* = 9. Differences were considered statistically significant at *p* < 0.05. Superscript letters (a, b, c, d, e) denote statistical differences between groups.

**Table 8:** Effect of different nutraceutical supplements on serum kidney functions and glucose level (mg/dL).

Groups	G1	G2	G3	G4	G5
BUN (Blood Urea Nitrogen)	22.5 ± 0.26 ^e^	51.2 ± 0.58 ^a^	39.2 ± 0.45 ^b^	26.1 ± 0.3 ^c^	23.9 ± 0.27 ^d^
Creatinine	0.56 ± 0.01 ^e^	1.59 ± 0.02 ^a^	0.86 ± 0.01 ^b^	0.80 ± 0.01 ^c^	0.76 ± 0.01 ^d^
Uric acid	1.46 ± 0.02 ^e^	3.39 ± 0.04 ^a^	2.84 ± 0.03 ^b^	2.3 ± 0.03 ^c^	2.02 ± 0.02 ^d^
Glucose	80.2 ± 0.91 ^d^	139.8 ± 1.6 ^a^	92.7 ± 1.1 ^b^	85.9 ± 0.98 ^c^	82.6 ± 0.94 ^d^

Results are presented as the mean ± SE, *n* = 9. Differences were considered statistically significant at *p* < 0.05. Superscript letters (a, b, c, d, e) denote statistical differences among groups.

**Table 9:** Effect of different nutraceutical supplements on total short-chain fatty acid (SCFA) concentration and SCFA profile of colonic content.

Groups	G1	G2	G3	G4	G5
Colonic content pH	6.87 ± 0.08 ^a^	6.93 ± 0.08 ^a^	6.84 ± 0.08 ^a^	6.29 ± 0.07 ^b^	6.07 ± 0.07 ^c^
Total SCFA concentration	139.4 ± 1.58 ^d^	176.2 ± 2.0 ^b^	171.2 ± 1.95 ^c^	207.1 ± 2.35 ^a^	212.1 ± 2.41 ^a^
Acetic acid	122.5 ± 1.39 ^c^	163.3 ± 1.86 ^b^	156.3 ± 1.78 ^b^	185.2 ± 2.11 ^a^	190.2 ± 2.16 ^a^
Propionic acid	11.0 ± 0.12 ^c^	8.9 ± 0.1 ^d^	10.6 ± 0.12 ^d^	16.7 ± 0.19 ^a^	15.9 ± 0.18 ^b^
Isobutyric acid	1.56 ± 0.02 ^c^	1.48 ± 0.02 ^d^	1.76 ± 0.02 ^b^	1.74 ± 0.02 ^b^	2.3 ± 0.03 ^a^
Butyric acid	3.29 ± 0.04 ^c^	3.56 ± 0.04 ^b^	3.2 ± 0.04 ^c d^	3.39 ± 0.04 ^c^	3.61 ± 0.04 ^a b^

Results are presented as the mean ± SE, *n* = 9. Differences were considered statistically significant at *p* < 0.05. Superscript letters (a, b, c, d) denote statistical differences between groups.

**Table 10:** Effect of different nutraceutical supplements on fecal lipids.

Groups	G1	G2	G3	G4	G5
Total lipids	22.7 ± 0.26 ^e^	21.0 ± 0.24 ^d^	27.9 ± 0.31 ^c^	30.6 ± 0.35 ^b^	32.1 ± 0.36 ^a^
Total cholesterol	1.67 ± 0.02 ^e^	1.15 ± 0.01 ^d^	3.49 ± 0.04 ^c^	5.35 ± 0.06 ^b^	5.78 ± 0.07 ^a^
Triglyceride	0.84 ± 0.01 ^e^	0.32 ± 0.004 ^d^	1.61 ± 0.01 ^c^	2.02 ± 0.02 ^b^	2.07 ± 0.02 ^a^

Results are presented as the mean ± SE, *n* = 9. Differences were considered statistically significant at *p* < 0.05. Superscript letters (a, b, c, d, e) denote statistical differences between groups.

## Data Availability

Data are contained within the article.
